# Prolonged extrapyramidal symptoms induced by long‐term, intermittent administration of low‐dose olanzapine along with metoclopramide for emesis: A case report

**DOI:** 10.1002/npr2.12277

**Published:** 2022-06-18

**Authors:** Shoko Sakamoto, Yasuhiko Deguchi, Sawako Uchida, Yoshiaki Itoh, Koki Inoue

**Affiliations:** ^1^ Department of Neuropsychiatry Osaka Metropolitan University Graduate School of Medicine Osaka Japan; ^2^ Department of Hepatology Osaka Metropolitan University Graduate School of Medicine Osaka Japan; ^3^ Department of Neurology Osaka Metropolitan University Graduate School of Medicine Osaka Japan

**Keywords:** chemotherapy, drug‐induced parkinsonism, extrapyramidal disorder, metoclopramide, olanzapine

## Abstract

**Background:**

Antipsychotics with dopamine (D2) receptor antagonism can be effective for emesis in cancer patients. Extrapyramidal symptoms (EPS) induced by typical antipsychotics can be exacerbated by other D2 receptor antagonists. We describe a case of persistent EPS induced by long‐term, intermittent administration of low‐dose olanzapine along with metoclopramide for emesis.

**Case Presentation:**

A 59‐year‐old pancreatic cancer patient underwent chemotherapy for 7 months. He was referred to the psychiatry department because of restlessness and insomnia. Although he did not have obvious depressive symptoms, he was anxious about the cancer treatment. For chemotherapy‐induced nausea, he had been prescribed 5 mg of olanzapine intermittently for 7 months. He had last used the drug 9 days before presenting it to us. Additionally, he received metoclopramide and palonosetron as antiemetics. We considered akathisia and cancer‐related anxiety/agitation as possible causes of restlessness and insomnia, and prescribed clonazepam. However, his symptoms worsened, resulting in hospitalization. We reconsidered his symptoms as cancer‐related anxiety/agitation and prescribed quetiapine. Although it was effective, he had tremors and was assessed by a neurologist. Considering the clinical manifestations of rigidity, postural reflex disorder, and a mask‐like face, we suspected drug‐induced parkinsonism and replaced quetiapine with biperiden on the next day, leading to his discharge after 2 weeks. He did not have symptom recurrence even after discontinuation of biperiden.

**Conclusions:**

Long‐term, intermittent administration of low‐dose antipsychotics with other antiemetics having D2 receptor antagonism can cause prolonged EPS. Especially in cancer patients, who often require polypharmacy, clinicians should consider exacerbated adverse effects due to drug interactions.

**Abbreviations**
EPS: extrapyramidal symptoms
CTZ: chemoreceptor trigger zone
CYP: cytochrome P450

## 1 INTRODUCTION

The annual incidence of cancer is rising to more than a million in Japan because of the aging population.^1^ Almost 50% of patients with cancer develop psychiatric disorders, such as depression, anxiety disorders, sleep disorders, and adjustment disorders. Approximately, 30% of these patients were reported to be treated with psychotropic drugs on admission.^2^ Additionally, 40%–70% of cancer patients experience nausea or vomiting and often take antipsychotics, such as dopamine (D2) receptor antagonists, for emesis.^3^ Acute extrapyramidal symptoms (EPS) can occur shortly after initiating antipsychotics, while tardive EPS is dose‐related, which can be observed several months after using the drugs.^4^ EPS induced by typical antipsychotics, such as levomepromazine and haloperidol, can be exacerbated by other D2 receptor antagonists like metoclopramide.^5^To date, there is no report of EPS induced by long‐term, intermittent low‐dose atypical antipsychotics and prolonged by other antiemetics having D2 receptor antagonism properties. Herein, we describe a case of persisting EPS followed by intermittent administration of low‐dose olanzapine for 7 months for emesis along with metoclopramide.

### 1.1 Case presentation

A 59‐year‐old Japanese man with pancreatic cancer undergoing chemotherapy for 7 months was referred to our psychiatry outpatient department due to restlessness and insomnia. He had no personal or familial psychiatric history. He had been prescribed hypnotic drugs for 2 weeks with no improvement, culminating in sickness absenteeism. He complained of anxiety about the pancreatic cancer treatment and stood up several times during the session; however, he did not have obvious depressive symptoms, such as depressed mood, loss of motivation or appetite, and anhedonia. He occasionally had upper limb tremors that disappeared while speaking, and he took short walks outside every few minutes (as reported by his wife), leading to insomnia.

On checking his medication history, we found that he had been receiving olanzapine 5 mg intermittently (for a few days every 2 weeks) for 7 months, with no adverse effects. The last dose was 9 days before the symptom onset. To prevent and treat chemotherapy‐induced nausea, he also received 15 mg/day of metoclopramide for 2 months, 0.75 mg of palonosetron, 6.6 mg of dexamethasone injection, 125 mg of aprepitant once at the start day of chemotherapy, and 80 mg of aprepitant twice (on the second and third day of chemotherapy), every 2 weeks. We considered differential diagnoses such as akathisia and cancer‐related anxiety/agitation and prescribed clonazepam 1.5 mg/day and brotizolam 0.25 mg/day. However, his restlessness and insomnia worsened, and he was prescribed Yokukansan and eperisone hydrochloride by his primary physician, which were ineffective. He was admitted to our hospital due to limb weakness and was referred to our department for further examination and treatment.

**FIGURE 1:**
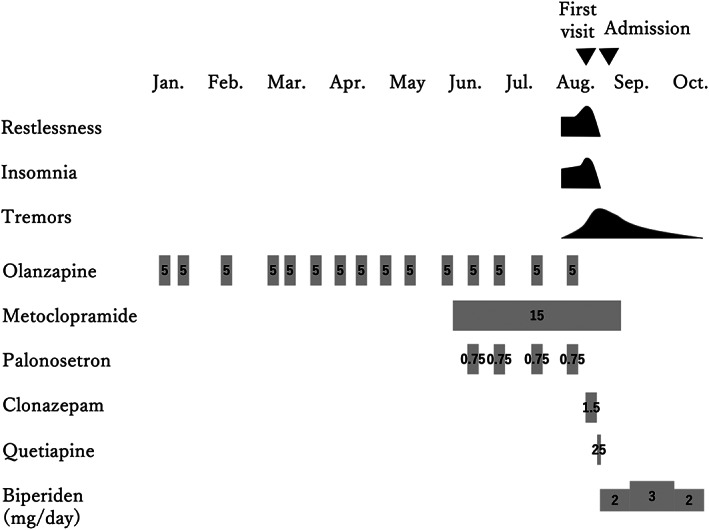
Clinical course of the patient with drug‐induced extrapyramidal symptoms

He exhibited restlessness. Blood biochemical examination showed no abnormalities. Since his symptoms did not subside following clonazepam administration, we considered cancer‐related anxiety/agitation, rather than simple akathisia, to be the cause of his symptoms and prescribed quetiapine once; subsequently, he was calm and slept well. However, his condition was still unstable, and was referred to the Neurology department on the next day after admission. Neurological findings such as tremors, rigidity, postural reflex disorder, and a mask‐like face were noted, and drug‐induced parkinsonism was strongly suspected with 21 points on the drug induced extra pyramidal symptoms scale (DIEPSS). We discontinued quetiapine and started biperiden (2 mg/day). On the next day, his akathisia and tremors almost disappeared. We diagnosed him with EPS induced by olanzapine and metoclopramide. His physician revised his chemotherapy regimen to TS‐1, a compounding agent of tegafur, gimeracil, and oteracil potassium, having a lower risk of nausea. Following the initiation of therapy using the new anticancer agent, he had no adverse effects and was discharged with 4 points on DIEPSS within 2 weeks after admission. Biperiden was discontinued 2 months later as his EPS did not recur. He resumed work and pancreatic cancer chemotherapy and had no recurrence of EPS for 4 months (Figure 1).

## 2 DISCUSSION AND CONCLUSIONS

We report a case of prolonged EPS induced by long‐term, intermittent administration of low‐dose olanzapine along with metoclopramide. Anticancer drugs can cause nausea by stimulating the vomiting center in the medulla oblongata through the serotonin 5‐HT3 receptor located in the upper gastrointestinal tract and the neurokinin‐1 and D2 receptors in the chemoreceptor trigger zone (CTZ); therefore, antagonists of serotonin, substance P, or dopamine are used as antiemetics. Antipsychotics that antagonize the D2 receptor in the CTZ can be effective for nausea.^6^ The effects and adverse effects (such as drowsiness and light‐headedness) of olanzapine, the only approved atypical antipsychotic for antiemetic in Japan, have been studied and compared to those of other antiemetics, and a low dose of olanzapine administered after supper instead of before sleep was found to prevent severe adverse effects and improve nausea.^7^ In our case, besides low‐dose olanzapine, the patient was receiving antiemetic therapy with palonosetron, aprepitant, and dexamethasone.

EPS are common adverse effects of antipsychotics because of D2 receptor antagonism. Acute EPS can be seen in a few minutes to weeks after administration, while tardive EPS can be caused by continuous or discontinuous administration of the drug for at least 3 months, which is directly related to the duration of administration and dose and is often treatment‐resistant once it occurs.^4^ Female sex, older age, and diabetes are known risk factors for EPS.^8^

Since patients with cancer often receive polypharmacy for primary treatment, drug interaction should be considered while prescribing antipsychotics to prevent or treat nausea/vomiting. Metoclopramide, a commonly used antiemetic, antagonizes the D2 receptor in the CTZ. A patient treated with metoclopramide and olanzapine was reported to develop a malignant syndrome, suggesting higher D2 receptor antagonism due to drug interaction.^9^ Another patient who was administered metoclopramide and paroxetine developed EPS, indicating cytochrome P450 (CYP) 2D6 inhibition by both drugs.^10^ Although there are some reports of EPS induced by metoclopramide alone, it was an acute reaction, at high‐dose therapy (normal dose: 30–40 mg/day), or following intravenous administration.^10^, ^11^ Here, although the patient did not have any risk factors for EPS (sex, age, or comorbidity), he developed lasting EPS induced by olanzapine and metoclopramide. Considering the duration of administration, dose, and improvement with biperiden therapy, we believe the symptoms were due to interactions between olanzapine and other antiemetics having D2 receptor antagonism properties, rather than tardive EPS. Both olanzapine and metoclopramide are D2 receptor antagonists, and metoclopramide, a CYP2D6 inhibitor, may delay olanzapine excretion, leading to prolonged EPS. Palonosetron, which is metabolized by CYP2D6 like olanzapine and metoclopramide, was not supposed to affect the excretion of other drugs because it is not a CYP2D6 inhibitor. As quetiapine was prescribed of 25 mg only once after the onset of symptoms, it probably did not induce EPS.

Long‐term, intermittent administration of low‐dose antipsychotics with other antiemetics having D2 receptor antagonism properties can cause prolonged EPS due to drug interactions such as delayed drug excretion or inhibition of drug metabolism. Especially in cancer patients who often receive polypharmacy for primary treatment, clinicians should consider adverse effects of antipsychotics, which can be affected by drug interaction, as a potential cause of EPS during chemotherapy.

## AUTHOR CONTRIBUTIONS

SS produced the initial draft and interpreted the case findings. YD, SU, YI, and KI critically revised the draft. All authors have read and approved the final manuscript.

## CONFLICT OF INTEREST

The authors declare no conflict of interest.

## ETHICAL APPROVAL

N/A.

## APPROVAL OF THE RESEARCH PROTOCOL BY AN INSTITUTIONAL REVIEW BOARD

N/A.

## INFORMED CONSENT

Written informed consent was obtained from the patient for publication of this case report.

## REGISTRY AND THE REGISTRATION NO. OF THE STUDY/TRIAL

N/A.

## ANIMAL STUDIES

N/A.

## Data Availability

Data sharing is not applicable to this article as no data sets were generated or analyzed during the current study.
